# Which glomerular filtration rate estimation equations should be used in youth with type 1 diabetes?

**DOI:** 10.1007/s00467-025-07057-w

**Published:** 2025-11-15

**Authors:** Radhe Shantha Kumar, R. Neil Dalton, M. Loredana Marcovecchio

**Affiliations:** 1 Department of Chemical Engineering and Biotechnology, University of Cambridge, Cambridge, UK; 2 Evelina London Children’s Hospital, London, UK; 3 Department of Paediatrics, University of Cambridge, Cambridge Biomedical Campus, Cambridge, UK; 4 Department of Paediatric Endocrinology and Diabetes, Cambridge University Hospitals NHS Foundation Trust, Cambridge, UK

**Keywords:** Equations, Glomerular filtration rate, Performance, Kidney disease, Type 1 diabetes, Youth

## Abstract

**Background:**

Diabetic kidney disease (DKD) is the primary cause of kidney failure in type 1 diabetes (T1D). Early identification of subclinical DKD is based on albuminuria and glomerular filtration rate (GFR). However, no GFR equations have been validated in youth with T1D. This study evaluates the performance of GFR equations in youth with T1D.

**Methods:**

We compared a direct measure of GFR (mGFR) via plasma clearance of exogenous inulin, with estimated GFR (eGFR). eGFR was calculated with the following equations: Full-Age Spectrum (FAS-height and FAS-age); revised Lund-Malmö (LM-Rev); European Kidney Function Consortium (EKFC); Chronic Kidney Disease Epidemiology Collaboration, 2009 (CKD-EPI 2009); CKD-EPI 2021 (revised CKD-EPI 2009 without race adjustment); CKD-EPI40 (revised CKD-EPI 2021 with age-adjusted creatinine); Chronic Kidney Disease in Children (CKiD); CKiD Under 25 (sex-dependent); CKiD Under 25 (age- and sex-dependent); and Improving Renal Complications in Adolescents with Type 2 Diabetes through Research (iCARE). Bland–Altman analysis estimated performance (bias and accuracy (P10 and P30)).

**Results:**

In total, 141 children and adolescents at a mean age of 13.5 ± 3.3 years (range 6.2–18.5) and diabetes duration 5.7 ± 1.6 years were included. Their mean serum creatinine was 45.6 ± 12.1 μmol/L (0.52 ± 0.14 mg/dL) and mGFR 142.3 ± 25.0 mL/min/1.73 m^2^. For the whole population, FAS-height (bias = 8.37 mL/min/1.73 m^2^; P30 = 85.1%) and CKiD (bias = 11.9 mL/min/1.73 m^2^; P30 = 81.6%) performed best. These equations also outperformed others in subgroups (females, age groups < 11 years and 11–15.9 years). In contrast, CKiD in males (bias = 5.23 mL/min/1.73 m^2^; P30 = 81.0%) and CKD-EPI 2009 in the 16–18.5 years subgroup (bias = −0.04 mL/min/1.73 m^2^; P30 = 84.6%) were top performers.

**Conclusions:**

Subgroups of children and adolescents with T1D may benefit from different eGFR equations for accurate subclinical DKD evaluation.

**Graphical abstract:**

**Supplementary Information:**

The online version contains supplementary material available at 10.1007/s00467-025-07057-w.

**Figure:**
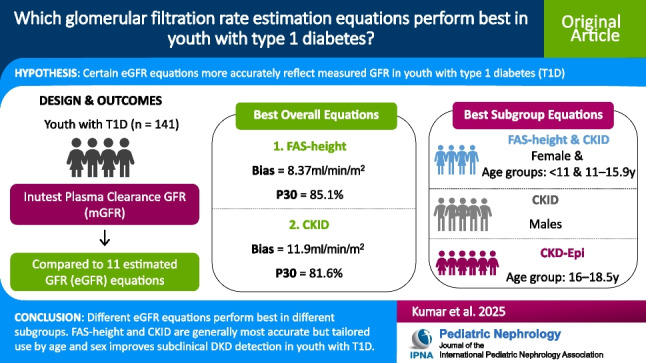
A higher resolution version of the Graphical abstract is available as Supplementary information.

## Introduction

Diabetic kidney disease (DKD) is a major microvascular complication of type 1 diabetes (T1D), and the leading cause of kidney failure, contributing to significant morbidity and mortality in children and adolescents [1]. DKD is characterised by progressive increases in urinary albumin excretion (albuminuria) and changes in glomerular filtration rates (GFR) [2]. While there are clear recommendations on how to assess albuminuria, monitoring GFR in routine clinical practice remains challenging [3, 4].

Although clearance of exogenous markers like inulin or iohexol is the gold standard for measuring GFR, these methods are impractical in clinical settings [5]. Consequently, clinical practice relies on estimated GFR (eGFR) equations based on endogenous circulating markers such as creatinine and/or cystatin C, and adjusting for variables such as age, sex, ethnicity, and height [6].

Identifying the most suitable equation(s) that provides GFR estimates closely aligned with directly measured GFR values is crucial to improve the detection of early changes in kidney function in T1D [5]. Although the Chronic Kidney Disease Epidemiology (CKD-EPI) equation has been widely used in adults, recent pooled data suggest it may not accurately estimate point GFR or track GFR decline in individuals with T1D [7, 8]. For the paediatric population, several GFR equations, incorporating factors like age, sex, weight, height, and disease category, have been proposed [9, 10]. However, most of these equations were developed and validated in cohorts of children with chronic kidney disease due to different causes, rather than in children and adolescents with T1D [9, 10]. To date, studies evaluating the validity of eGFR equations specifically in children and adolescents with T1D remain limited. A recent study by Gaebe et al. proposed that the sex-adjusted Chronic Kidney Disease in Children (CKiD1) equation exhibited superior performance in estimating GFR in adolescents and young adults with T1D [11]. However, this study focused solely on individuals aged 17–26 years, leaving uncertainties regarding its applicability to younger populations under 17 years of age [11]. Furthermore, a large-scale study by Boettcher et al., which analysed data from 36,782 adolescents with T1D, highlighted the lack of reliable GFR equations applicable across various subgroups of this population [12]. The aim of the present study was to assess the performance of both established and recently developed eGFR equations in a cohort of youth with T1D, and explore potential differences based on age and sex categories.

## Methods

### Study population

The study population comprised a subset of children and adolescents previously enrolled and followed up in the Oxford Regional Prospective Study (ORPS), with available data on a direct measurement of GFR at 5 years of diabetes duration. The characteristics of the ORPS cohort are detailed in previous publications [2, 13]. Briefly, children under 16 years of age residing in the Oxford Health Authority region were recruited soon after T1D diagnosis between 1986 and 1997. By the end of 1997, a total of 527 individuals had been recruited. Assessments were conducted at the end of the first year post-diagnosis, and annually thereafter. Ethical approval was obtained from the Oxford ethics committee (Rec No. 00\5\65); written consent was obtained from the parents and consent from study participants.

### Procedures

The annual assessment encompassed various parameters, including height, weight, body mass index (BMI), and blood samples. A direct measure of GFR (mGFR) was performed at a 5-year diabetes duration [14].

HbA1c was analysed in a central laboratory [13], and values, originally measured in per cent, were converted to mmol/mol by subtracting 2.15 and multiplying the result by 10.929. Plasma creatinine was measured by stable isotope dilution electrospray mass spectrometry-mass spectrometry (MSMS), a method traceable to the international reference standard (isotope dilution mass spectrometry, IDMS) and compliant with recommendations from the International Federation of Clinical Chemistry and Laboratory Medicine (IFCC) [13, 14]. Results were calculated using Analyst version 1.4.1. The direct measurement of GFR (mGFR) involved a single intravenous bolus of exogenous polyfructosan inulin (Inutest, Laevosan-Gesellschaft, Linz, Austria), a branched-chain compound with a Stokes radius profile equivalent to inulin [2]. The subsequent plasma clearance of exogenous inulin was estimated through numerical analysis using a concentration-dependent time curve as previously reported [13, 14]. In most participants, venous access was obtained in both arms, allowing blood sampling at multiple time points (e.g. 5, 60, 120, 150, and 180 min) after a single intravenous bolus of exogenous inulin [2]. GFR was calculated using a Simplex curve-fitting procedure applied to the plasma concentration–time data, followed by convolution with a theoretical continuous input function (a series of unit impulses), which allowed modelling of both the distribution and elimination phases [2]. In a subset of participants, however, venous access could only be obtained in one arm, and the same cannula was used for both injection and sampling [2]. Despite careful flushing, contamination of early samples (e.g. 5 min) could not be excluded. For these cases, GFR was estimated using a mono-exponential model applied to the elimination-phase data (120–180 min). A validated correction factor (×0.68), derived from comparisons with full double-compartment curves in the cohort [2], was then applied to ensure comparable GFR estimates between methods.

### eGFR calculation

GFR was estimated using 11 equations: Full-Age Spectrum (FAS-height [15], FAS-age [16]), revised Lund-Malmö (LM-Rev) [17], European Kidney Function Consortium (EKFC) [18], Chronic Kidney Disease Epidemiology Collaboration 2009 (CKD-EPI 2009) [19], CKD-EPI 2021 (revised CKD-EPI 2009 without race adjustment) [20], CKD-EPI40 (revised CKD-EPI 2021 with age-adjusted creatinine) [10], Chronic Kidney Disease in Children (CKiD) [9], CKiD Under 25 (sex-dependent) [21], CKiD Under 25 (age- and sex-dependent) [21], and Improving Renal Complications in Adolescents with Type 2 Diabetes through Research (iCARE) [22].

These equations were selected based on their applicability to the paediatric and young adult age range, their evaluation in populations with diabetes and chronic kidney disease, and their ability to estimate GFR across a range of kidney function, particularly in the higher GFR range typical of early DKD. These equations are further detailed in Supplementary Table S1.

### Statistical analysis

The performance of each GFR equation is reported as bias, precision, and accuracy. Bias is the difference between estimated GFR and measured GFR (eGFR–mGFR), calculated as the mean over all the differences. Precision is the standard deviation (SD) of bias, and accuracy is the percentage of eGFR values within 10% (P10) and 30% (P30) of the measured GFR. The reference interval corresponds to the values between the 2.5th and 97.5th percentiles of the distribution of the results.

Additionally, the data are depicted through Bland–Altman plots, wherein the difference between measured GFR and estimated GFR is plotted against the mean of measured GFR and estimated GFR. Hyperfiltration was defined as GFR ≥ 135 mL/min/1.73 m^2^ [23].

For subgroup analyses, age categories were defined as < 11 years, 11–15.9 years, and 16–18.5 years. These cutoffs reflect childhood, early to mid-adolescence, and late adolescence, aligning with developmental stages that may influence kidney function and eGFR estimates. Statistical analyses were conducted using GraphPad Prism version 9.4.1 for Mac (GraphPad Software). Data are presented as mean ± SD, unless stated otherwise.

## Results

### Baseline characteristics

A total of 141 children and adolescents diagnosed with T1D were included in the present analysis (male, *n* = 79). Baseline characteristics of the study population are reported in Table 1. The mean age at the time of GFR measurements was 13.5 ± 3.3 years (range 6.2–18.5 years), with a diabetes duration of 5.7 ± 1.6 years; mean BMI was 20.9 ± 3.6 kg/m^2^ (BMI *z*-score: 0.71 ± 0.91), and HbA1c level was 10.1 ± 1.8% (87.0 ± 19.9 mmol/mol) (Table 1). The mean plasma creatinine was 45.6 ± 12.1 μmol/L (0.52 ± 0.14 mg/dL), and the mean mGFR was 142.3 ± 25.0 mL/min/1.73 m^2^. Eighty-three participants (58.9%) met the criteria for hyperfiltration, whereas none had a GFR < 90 mL/min/1.73 m^2^.

**Table 1:** Baseline characteristics of the study population

Characteristic	Total population (*n* = 141)	Male (*n* = 79)	Female (*n* = 62)
Age (years)	13.5 ± 3.3	13.6 ± 3.2	13.5 ± 3.3
Diabetes duration (years)	5.7 ± 1.6	5.6 ± 1.6	6.0 ± 2.0
BMI (kg/m^2^)	20.9 ± 3.6	20.9 ± 3.6	20.9 ± 3.6
BMI *z*-score	0.71 ± 0.91	0.70 ± 0.90	0.72 ± 0.92
HbA1c %	10.1 ± 1.82	10.0 ± 1.81	10.1 ± 1.83
HbA1c (mmol/mol)	87.0 ± 19.9	86.0 ± 19.7	87.0 ± 20
Creatinine, μmol/L (mg/dL)	45.6 ± 12.1 (0.52 ± 0.14)	45.9 ± 12.04 (0.52 ± 0.14)	45.6 ± 12.1 (0.52 ± 0.14)
mGFR (mL/min/1.73 m^2^)	142.3 ± 25.0	142.6 ± 24.1	142.3 ± 25.0
mGFR ≤ 90 mL/min/1.73 m^2^	0	0	0
mGFR ≥ 135 mL/min/1.73 m^2^	83 (58.9)	51 (36.2)	34 (24.1)

Data are shown as mean ± SD or *n* (%). Creatinine values in mg/dL were derived using the conversion: 1 mg/dL = 88.4 μmol/L

*BMI*, body mass index; *HbA1c*, glycated haemoglobin; *mGFR*, measured glomerular filtration rate

### eGFR equations vs. mGFR

For the entire study population, FAS-height and CKiD were identified as the equations with the least bias, with values of 8.37 ± 30.79 mL/min/1.73 m^2^ and 11.87 ± 31.51 mL/min/1.73 m^2^, respectively (Table 2). In contrast, iCARE and CKD-EPI40 demonstrated the highest bias, with values of 31.50 ± 30.58 mL/min/1.73 m^2^ and 29.75 ± 26.71 mL/min/1.73 m^2^, respectively. The Bland–Altman analysis of the 11 eGFR equations is presented in Supplementary Figures S1–6.

**Table 2:** eGFR equations bias, 95% confidence intervals (CI), and percentage of eGFR within 10% (P10) and 30% (P30) of mGFR for the entire study population

Method	Mean GFR	Bias	SD bias	95% CI	P10 (%)	P30%
mGFR	142.26 ± 25.03					
FAS-height	133.89 ± 24.59	8.37	30.79	−51.97; 68.71	37.58	85.10
FAS-age	129.73 ± 26.63	12.53	32.89	−51.92; 76.99	29.78	81.56
LM-Rev	114.20 ± 0.78	28.06	24.25	−19.46; 75.58	27.65	77.30
CKD-EPI 2009	156.48 ± 18.82	−14.21	27.70	−68.50; 40.07	36.88	78.72
CKD-EPI 2021	162.05 ± 18.70	−19.78	28.52	−75.68; 36.11	32.62	71.63
CKD-EPI40	112.52 ± 15.78	29.75	26.71	−22.60; 82.09	20.56	72.34
CKiD	130.39 ± 25.66	11.87	31.51	−49.90; 73.64	31.91	81.56
CKiD Under 25 (sex-dependent)	125.81 ± 23.91	16.45	29.30	−40.97; 73.88	34.75	82.27
CKiD Under 25 (age- and sex-dependent)	123.32 ± 23.13	18.94	29.52	−38.91; 76.80	32.62	80.14
EKFC	114.76 ± 8.24	27.50	24.43	−20.37; 75.38	26.95	50.35
iCARE	110.56 ± 19.30	31.50	30.58	−28.44; 91.44	17.02	68.38

*EKFC*, European Kidney Function Consortium; *CKD-EPI*, Chronic Kidney Disease Epidemiology Collaboration; *CKiD*, Chronic Kidney Disease in Children; *FAS*, Full-Age Spectrum; *iCARE*, Improving renal Complications in Adolescents with T2D through REsearch; *LM-Rev*, revised Lund-Malmö; *mGFR*, measured GFR

Furthermore, we undertook a subgroup analysis in the following groups:

(i) Based on sex: In females (Table 3a), the equations with the least bias were FAS-height (−0.43 ± 31.31 mL/min/1.73 m^2^) and CKi2D (−0.37 ± 30.84 mL/min/1.73 m^2^). Conversely, within the male cohort (Table 3b), the equation displaying the lowest bias was CKiD (5.23 ± 30.62 mL/min/1.73 m^2^). The equations with the highest bias were iCARE (32.36 ± 29.02 mL/min/1.73 m^2^) in females, and CKD-EPI40 (34 ± 27.75 mL/min/1.73 m^2^) in males. (ii) Based on age groups: under 11 years, 11–15.9 years, and 16–18.5 years. In the under 11 years group, the equations demonstrating the least bias were FAS-height (8.57 ± 31.75 mL/min/1.73 m^2^), CKiD Under 25 (age- and sex-dependent) (8.66 ± 27.62 mL/min/1.73 m^2^), and CKiD (13.95 ± 32.63 mL/min/1.73 m^2^) (Table 4a). Similarly, in the 11–15.9 years group, FAS-height (8.19 ± 28.28 mL/min/1.73 m^2^), CKiD (10.65 ± 28.48 mL/min/1.73 m^2^), and CKiD Under 25 (sex-dependent) (14.31 ± 26.93 mL/min/1.73 m^2^) exhibited the lowest bias (Table 4b). In the 16–18.5 years group, the top-performing equations were CKD-EPI 2009 (−0.04 ± 24.74 mL/min/1.73 m^2^) and CKD-EPI 2021 (−5.26 ± 26.63 mL/min/1.73 m^2^) (Table 4c). Notably, LM-Rev, CKD-EPI40, iCARE, and EKFC showed relatively poorer performance across all age subgroups.
  **Table 3:** Summary of equation bias, 95% confidence intervals and mean GFR for each equation in females (*n*=62; 3a) and males (*n*= 79; 3b)

A						
Method	Mean GFR	Bias	SD Bias	95% CI	P10%	P30%
mGFR	142.26±25.03					
FAS-height	133.89±24.59	−0.43	31.31	−61.79; 60.93	37.09	87.09
FAS-age	129.73±26.63	−1.47	32.87	−65.91; 62.96	30.65	83.87
LM-Rev	114.20±10.78	25.22	24.32	−22.44; 72.88	24.19	79.03
CKD-EPI 2009	156.48±18.82	−13.75	26.44	−65.56; 38.06	40.32	80.65
CKD-EPI 2021	162.05±18.70	−26.46	26.92	−79.22; 26.29	30.65	64.52
CKD-EPI40	112.52±15.78	24.33	24.48	−23.65; 72.31	38.70	90.32
CKiD	130.39±25.66	−0.37	30.84	−60.82; 60.08	32.26	82.26
CKiD Under 25 (sex-dependent)	125.81±23.91	11.98	29.41	−45.67; 69.62	32.26	82.26
CKiD Under 25 (age- and sex-dependent)	123.32±23.13	13.48	30.78	−46.85; 73.80	30.65	80.65
EKFC	114.76±8.24	22.15	24.10	−25.08; 69.39	25.81	82.26
iCARE	110.56±19.30	32.36	29.02	−24.51; 89.24	17.74	64.52
B						
Method	Mean GFR	Bias	SD Bias	95%CI	P10%	P30%
mGFR	142.56±24.15					
FAS-height	133.77±24.66	15.27	28.72	−41.02; 71.57	37.97	83.54
FAS-age	129.54±26.73	23.53	28.59	−32.51; 79.57	29.11	79.75
LM-Rev	114.26±10.86	30.29	24.11	−16.96; 77.55	30.38	75.95
CKD-EPI 2009	156.18±18.81	−14.58	28.81	−71.04; 41.89	34.18	77.22
CKD-EPI 2021	161.56±18.48	−14.54	28.81	−71.00; 41.92	34.18	77.22
CKD-EPI40	112.46±15.93	34.00	27.75	−20.38; 88.38	39.24	89.87
CKiD	129.99±24.09	5.23	30.62	−54.74; 65.29	31.65	81.01
CKiD Under 25 (sex-dependent)	125.60±23.94	19.96	28.91	−36.70; 76.63	21.52	69.62
CKiD Under 25 (age- and sex-dependent)	123.1523.17	24.24	27.94	−31.52; 77.99	34.18	79.75
EKFC	114.73±8.30	31.70	24	−15.35; 78.74	27.85	74.68
iCARE	111.14±19.01	31.18	29.64	−26.92; 89.27	17.72	69.62
  **Table 4:** Summary of equation bias, 95% confidence intervals, and mean GFR for each equation by age groups: < 11-year (n = 37, 4a); 11–15.9 years (n = 61; 4b); 16–18.5 years (n = 43; 4c)

A						
Method	Mean GFR	Bias	SD Bias	95% CI	P10%	P30%
mGFR	142.26±25.03					
FAS-height	133.89±24.59	8.57	31.75	−53.67; 70.81	37.84	91.89
FAS-age	129.73±26.62	13.68	34.11	−53.17; 80.53	35.14	81.08
LM-Rev	114.20±10.77	30.68	24.49	−17.33; 78.68	24.32	72.97
CKD-EPI 2009	156.48±18.82	−11.06	28.90	−67.70; 45.59	27.03	67.57
CKD-EPI 2021	162.05±18.07	−17.17	30.01	−75.98; 41.65	21.62	59.46
CKD-EPI40	112.52±15.78	20.95	25.78	−29.58; 71.49	45.95	86.49
CKiD	130.39±25.66	13.95	32.63	−54.88; 70.41	29.73	83.78
CKiD Under 25 (sex-dependent)	125.81±23.91	8.66	27.62	−45.47; 62.79	32.43	86.49
CKiD Under 25 (age- and sex-dependent)	123.32±23.13	21.29	30.44	−38.37; 80.95	37.84	89.19
EKFC	114.76±8.24	29.78	26.14	−21.46; 81.02	21.62	70.27
iCARE	110.56±19.30	32.66	30.95	−27.99; 93.32	2.70	24.32
B						
Method	Mean GFR	Bias	SD Bias	95% CI	P10%	P30%
mGFR	143.90± 23.19					
FAS-height	134.10± 24.79	8.19	28.28	−47.24; 63.63	39.34	88.52
FAS-age	129.8± 26.61	11.60	30.14	−47.48; 70.67	31.15	88.52
LM-Rev	114.48± 10.84	25.38	22.73	−19.17; 69.93	31.15	80.33
CKD-EPI 2009	156.76± 18.76	−14.43	23.99	−61.45; 32.60	39.34	80.33
CKD-EPI 2021	162.12± 18.37	−19.08	23.99	−66.10; 27.93	34.43	72.13
CKD-EPI40	112.78± 15.82	27.18	23.06	−18.03; 72.38	40.98	95.08
CKiD	130.54± 25.51	10.65	28.48	−45.17; 66.48	36.07	88.52
CKiD Under 25 (sex-dependent)	126.17± 23.83	14.31	26.93	−38.47; 67.09	29.51	78.69
CKiD Under 25 (age- and sex-dependent)	123.60± 23.60	17.68	26.66	−34.59; 69.94	34.43	81.97
EKFC	114.85± 8.38	26.28	23.05	−18.89; 71.45	32.79	81.97
iCARE	110.96± 19.24	27.95	23.49	−18.08; 73.99	19.67	78.69
C						
Method	Mean GFR	Bias	SD Bias	95% CI	P10%	P30%
mGFR	141.47±23.28					
FAS-height	133.66±24.88	6.85	36.65	−64.98; 78.68	35.90	76.92
FAS-age	129.45±26.98	14.00	40.11	−64.61; 92.61	25.64	71.79
LM-Rev	114.07±10.89	30.24	25.64	−20.01; 80.49	28.21	79.49
CKD-EPI 2009	155.99±18.92	−0.04	24.74	−48.52; 48.44	43.59	84.62
CKD-EPI 2021	161.49±18.62	−5.26	26.63	−57.45; 46.94	28.21	69.23
CKD-EPI40	112.22±15.98	36.06	29.77	−22.29; 94.40	30.23	76.74
CKiD	129.85±25.77	21.64	35.59	−48.10; 91.39	28.21	69.23
CKiD Under 25 (sex-dependent)	125.32±24.05	26.20	31.85	−36.22; 88.62	16.28	55.81
CKiD Under 25 (age- and sex-dependent)	122.89±23.29	24.27	34.64	−43.61; 92.16	28.21	71.79
EKFC	114.61±8.35	27.26	25.25	−22.22; 76.75	25.64	82.05
iCARE	110.97±19.18	15.50	25.48	−34.43; 65.44	25.64	89.74

*EKFC*, European Kidney Function Consortium; *CKD-EPI*, Chronic Kidney Disease Epidemiology Collaboration; *CKiD*, Chronic Kidney Disease in Children; *FAS*, Full-Age Spectrum; *iCARE*, Improving renal Complications in Adolescents with T2D through REsearch; *LM-Rev*, revised Lund-Malmö; *mGFR*, measured GFR

## Discussion

Accurate and non-invasive assessment of DKD in youth with T1D is limited by the lack of eGFR equations specifically validated in this population [12]. Most existing formulas were developed in paediatric cohorts with chronic kidney disease or in adults, and their applicability to children and adolescents with T1D remains uncertain. This gap has led to variations in clinical practice, with no clear consensus on which equations are most appropriate across paediatric subgroups [12]. To address this, we evaluated the performance of 11 eGFR equations in a cohort of 141 children and adolescents with T1D across the whole population, and the FAS-height and CKiD equations demonstrated the best overall performance, with subgroup analyses revealing notable sex-specific differences in equation performance. For females, both FAS-height and CKiD exhibited minimal bias, whereas in males, CKiD alone outperformed the other equations. These findings differ from those of Gaebe et al., who identified the CKiD Under 25 (sex-dependent) equation as superior to the original CKiD equation in adolescents and young adults with T1D [11]. However, their study was performed in an older cohort (17–26 years) and focused on five eGFR equations [11]. In contrast, our study spanned a broader age range (6.2–18.5 years) and validated 11 eGFR equations.

Additionally, age-based subgroup analyses show that FAS-height, CKiD, and CKiD Under 25 (sex-dependent) equations demonstrate the best performance in youth younger than 16 years, whereas CKD-EPI 2009 and CKD-EPI 2021 perform better in the 16–18.5 years subgroup. This suggests that formulas currently in use in adults, such as CKD-EPI 2009 and CKD-EPI 2021, could be applied starting at 16 years of age. Our findings support the utility of the FAS-height and CKiD formulas for estimating eGFR in children and younger adolescents with T1D, where height is a key determinant. These observations are consistent with earlier studies, including the original Schwartz formula, which identified height as the most influential anthropometric factor for eGFR estimation in paediatric populations [9]. The iCARE equation, originally derived in adolescents with obesity and type 2 diabetes (T2D), was included to evaluate its performance outside its development cohort [22]. Given the distinct clinical and metabolic profiles of youth with T2D and T1D, interpretation of its results should be made with caution. Nevertheless, its inclusion provides a relevant comparison and highlights the potential limitations of applying population-specific equations more broadly.

Our results also contradicted earlier findings in children and adolescents aged 2–17 years [10], where the CKD-EPI40 led to extensive improvements in bias, precision, and accuracy. In our study, CKD-EPI40 performed poorly across most subgroup analyses. These differences may be attributed to variations in study populations and GFR ranges and highlight the need to tailor evaluations to specific subgroups.

A limitation of this study is that the cohort largely comprised children and adolescents with early T1D kidney disease, characterised primarily by increased GFR, and did not include participants with a low GFR, where most proposed eGFR formulas were previously tested. Accordingly, the findings are applicable only to the normal/hyperfiltration stage of T1D kidney disease and cannot be extrapolated to individuals with impaired kidney function or more advanced DKD. In our historical cohort, HbA1c was high and this may have contributed to hyperfiltration and altered kidney haemodynamics [24]. Other limitations were the relatively small sample size and lack of ethnic diversity, as the cohort was predominantly White British. These factors limit the generalisability of our findings to more heterogeneous populations. Additionally, although combined creatinine-cystatin C equations have shown promise in improving accuracy [25], only creatinine-based equations were used in this study due to the unavailability of cystatin C measurements.

Despite these limitations, the study provides valuable insights into eGFR assessment in children and adolescents with T1D, particularly in the early stages of DKD, where elevated GFR (hyperfiltration) is common and may precede traditional clinical markers such as albuminuria. By evaluating a wide range of equations across a broad paediatric T1D age spectrum, it highlights the need for tailored approaches for eGFR assessment that account for age and sex to improve its clinical applicability.

## Conclusion

Our findings suggest that no single eGFR equation is universally applicable across the full spectrum of kidney function in youth with T1D, particularly in the context of early kidney disease characterised by hyperfiltration. While FAS-height and CKiD equations demonstrated relatively better performance in this cohort, these results do not allow selection of a best formula for hyperfiltrating T1D. Instead, equation performance is likely to vary across subgroups defined by age, sex, and the level of kidney function. In particular, equations such as CKiD Under 25 (age- and sex-dependent), which performed poorly at high GFR, may prove more suitable in youth with reduced kidney function, as they were developed in populations with mild to moderate CKD. Further studies including children and adolescents with impaired GFR are therefore essential to determine the most accurate equations in more advanced stages of T1D kidney disease. Such work should incorporate repeated direct measurements of GFR and evaluate the role of combined creatinine-cystatin C equations to improve accuracy across the full spectrum of kidney function.

## Supplementary Information

Below is the link to the electronic supplementary material.

ESM 1 Graphical abstract (120 KB)

ESM 2 (DOCX 1.33 MB) 

ESM 3 (DOCX 30.6 KB) 

## Data Availability

The datasets generated and analysed during the current study are available from the corresponding author on reasonable request.
