# Supplementary material for: Which glomerular filtration rate estimation equations should be used in youth with type 1 diabetes?
Source: Pediatr Nephrol. 2025 Nov 15;41(3):801–8. doi: 10.1007/s00467-025-07057-w (PMC12852128; doi:10.1007/s00467-025-07057-w)
Supplement: Supplementary file 1 — Graphical abstract (120 KB) [file 467_2025_7057_MOESM1_ESM.pptx]

## Slide 1
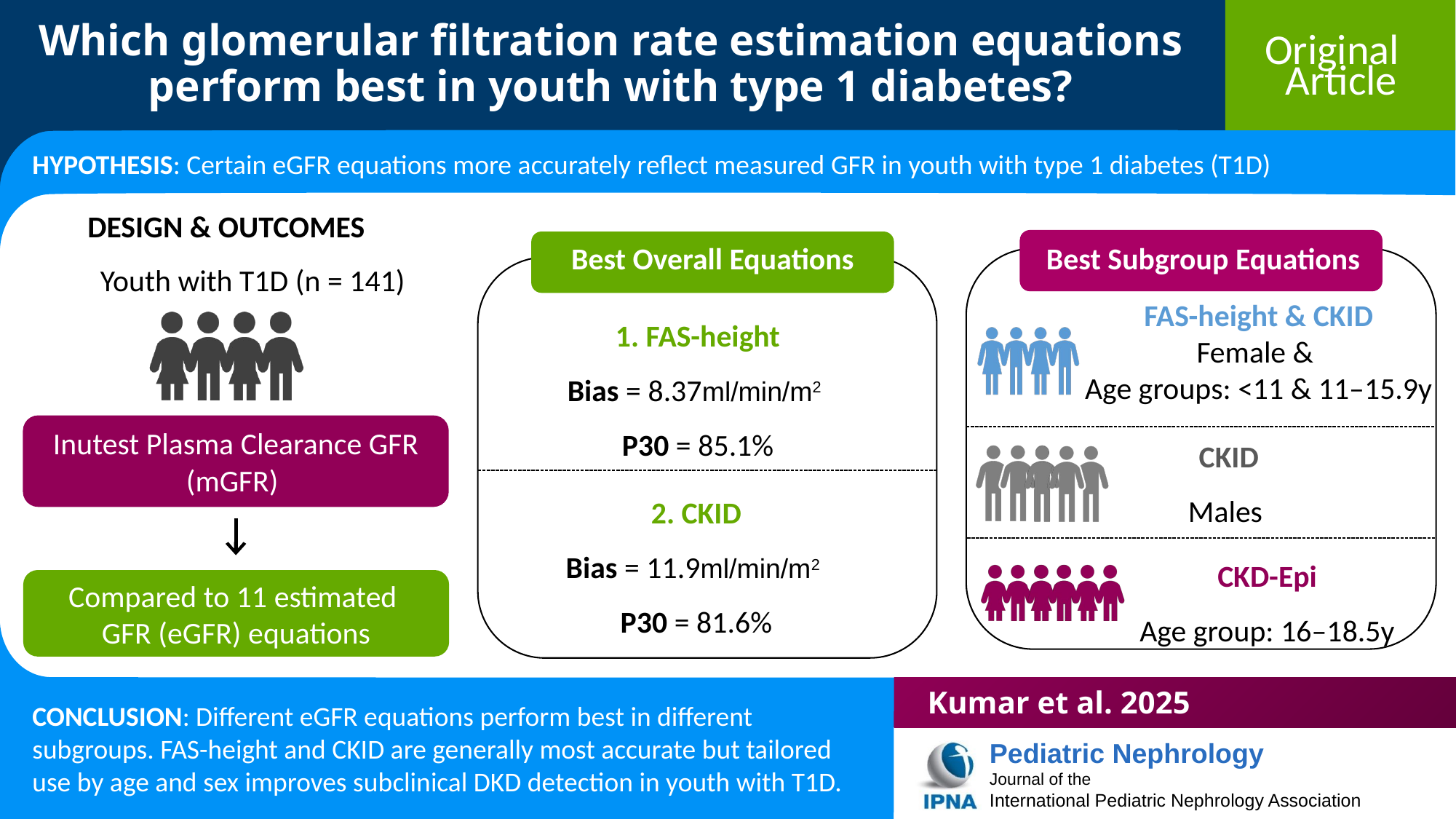

Which glomerular filtration rate estimation equations perform best in youth with type 1 diabetes?
HYPOTHESIS: Certain eGFR equations more accurately reflect measured GFR in youth with type 1 diabetes (T1D)
DESIGN & OUTCOMES
Best Subgroup Equations
Best Overall Equations
Youth with T1D (n = 141)
FAS-height & CKID
Female &
Age groups: <11 & 11–15.9y
1. FAS-height
Bias = 8.37ml/min/m2
P30 = 85.1%
CKID
Males
Inutest Plasma Clearance GFR (mGFR)
2. CKID
Bias = 11.9ml/min/m2
P30 = 81.6%
CKD-Epi
Age group: 16–18.5y
Compared to 11 estimated
GFR (eGFR) equations
Kumar et al. 2025
CONCLUSION: Different eGFR equations perform best in different subgroups. FAS-height and CKID are generally most accurate but tailored use by age and sex improves subclinical DKD detection in youth with T1D.
