# Supplementary material for: Which glomerular filtration rate estimation equations should be used in youth with type 1 diabetes?
Source: Pediatr Nephrol. 2025 Nov 15;41(3):801–8. doi: 10.1007/s00467-025-07057-w (PMC12852128; doi:10.1007/s00467-025-07057-w)
Supplement: Supplementary file 2 — (DOCX 1.33 MB) [file 467_2025_7057_MOESM2_ESM.docx]

**Supplementary Figures**

**
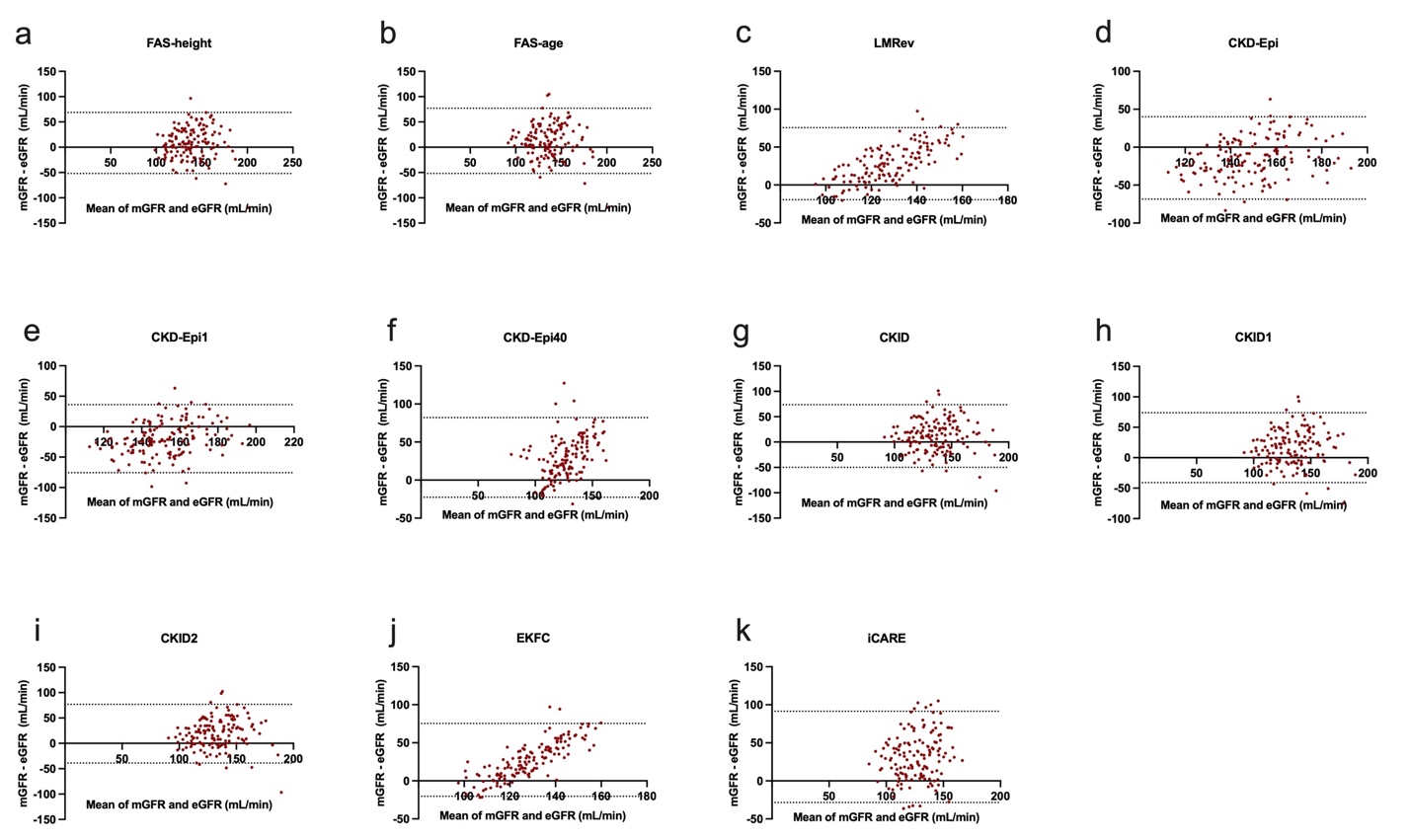
**

**Supplementary Figure S1.** Bland Altman plots for the whole study population

CDK-EPI: Chronic Kidney Disease Epidemiology; CKiD: Chronic Disease in Children; EKFC: European Kidney Function Consortium; FAS: Full-age spectrum; iCARE: Improving Renal Complications in Adolescents with Type 2 Diabetes through Research; LM-Rev: Lund-Malmo-revised; mGFR: measured glomerular filtration rate; eGFR: estimated glomerular filtration rate


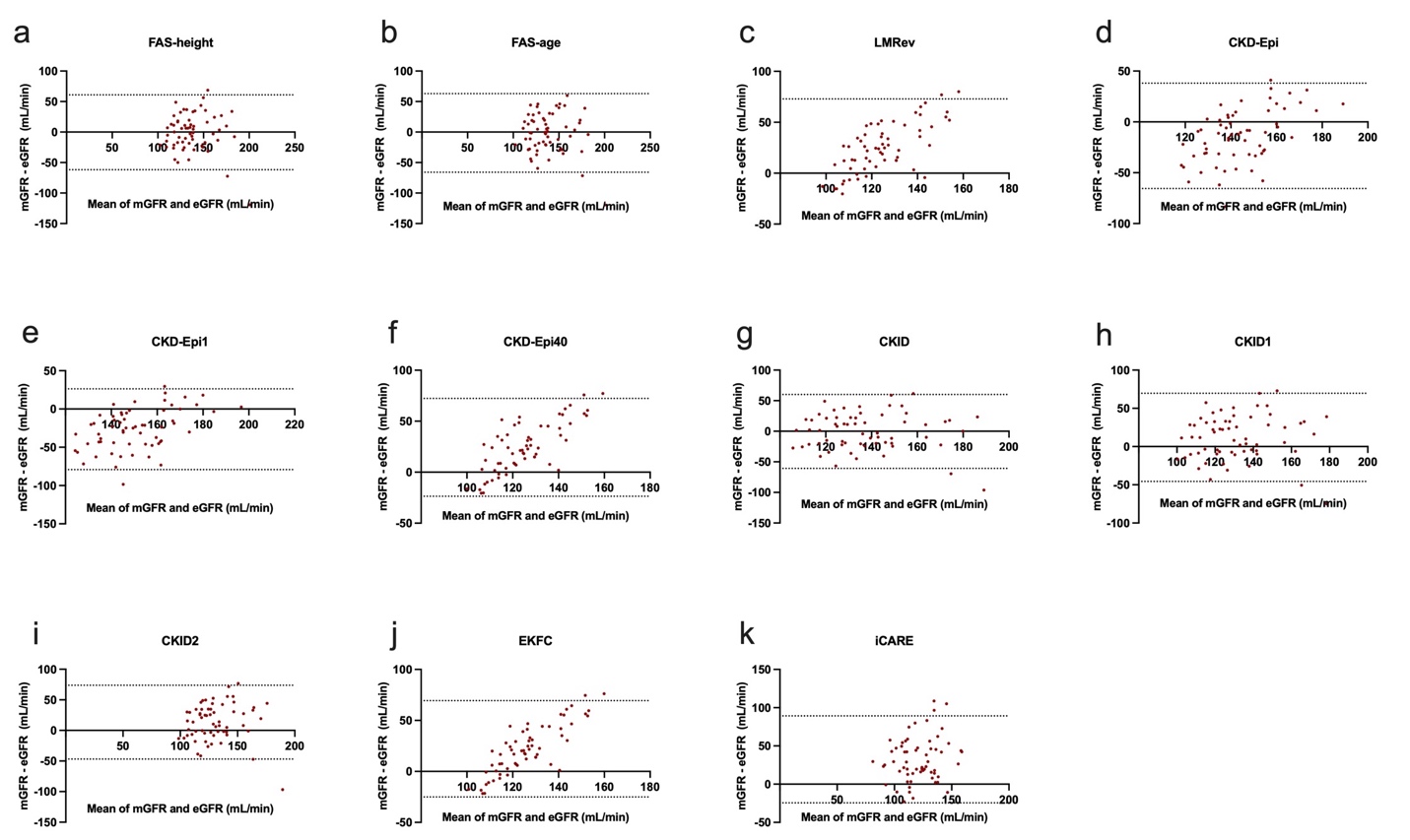


**Supplementary Figure S2.** Bland Altman plots for the female subgroup

CDK-EPI: Chronic Kidney Disease Epidemiology; CKiD: Chronic Disease in Children; EKFC: European Kidney Function Consortium; FAS: Full-age spectrum; iCARE: Improving Renal Complications in Adolescents with Type 2 Diabetes through Research; LM-Rev: Lund-Malmo-revised; mGFR: measured glomerular filtration rate; eGFR: estimated glomerular filtration rate


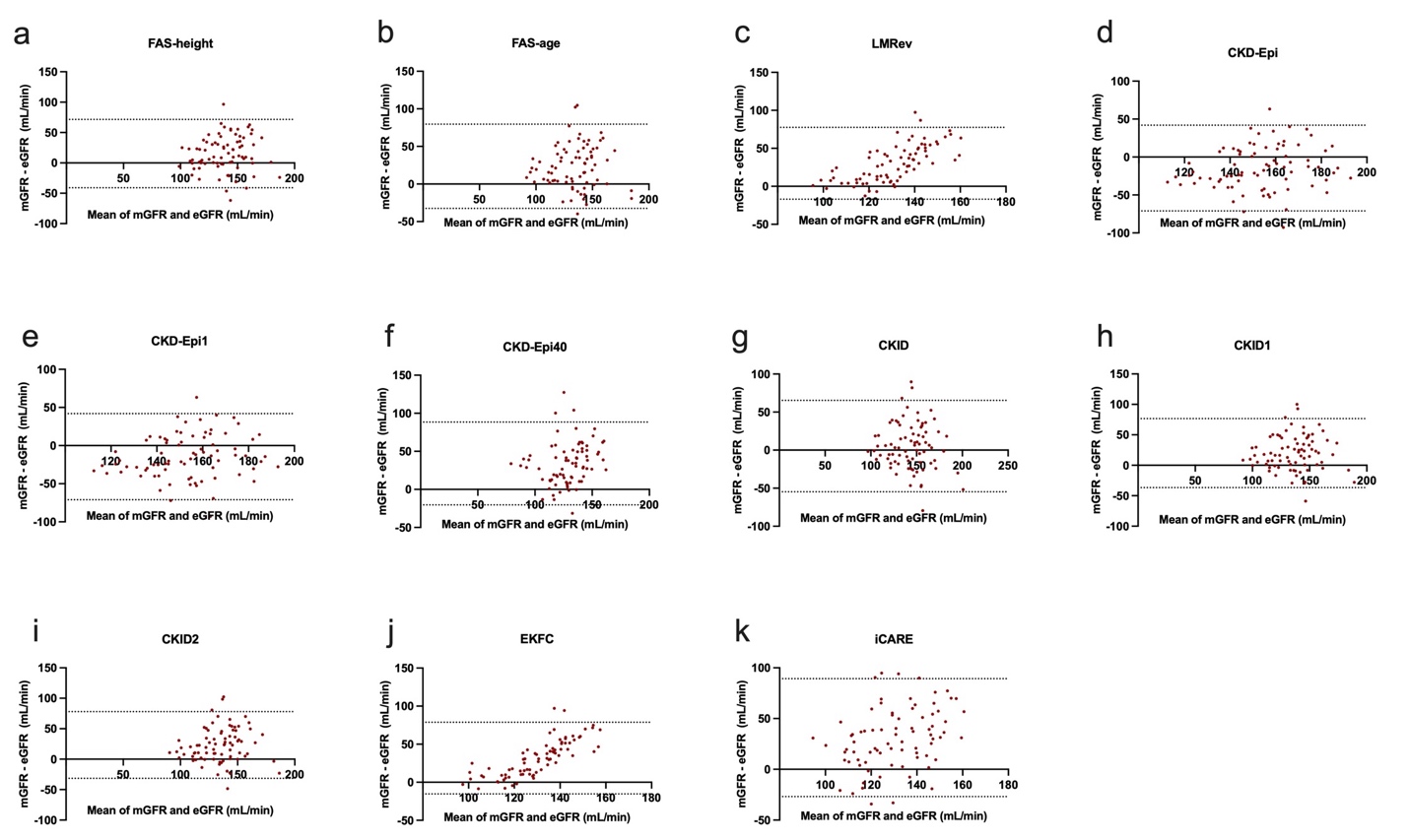


**Supplementary Figure S3.** Bland Altman plots for the male subgroup

CDK-EPI: Chronic Kidney Disease Epidemiology; CKiD: Chronic Disease in Children; EKFC: European Kidney Function Consortium; FAS: Full-age spectrum; iCARE: Improving Renal Complications in Adolescents with Type 2 Diabetes through Research; LM-Rev: Lund-Malmo-revised; mGFR: measured glomerular filtration rate; eGFR: estimated glomerular filtration rate


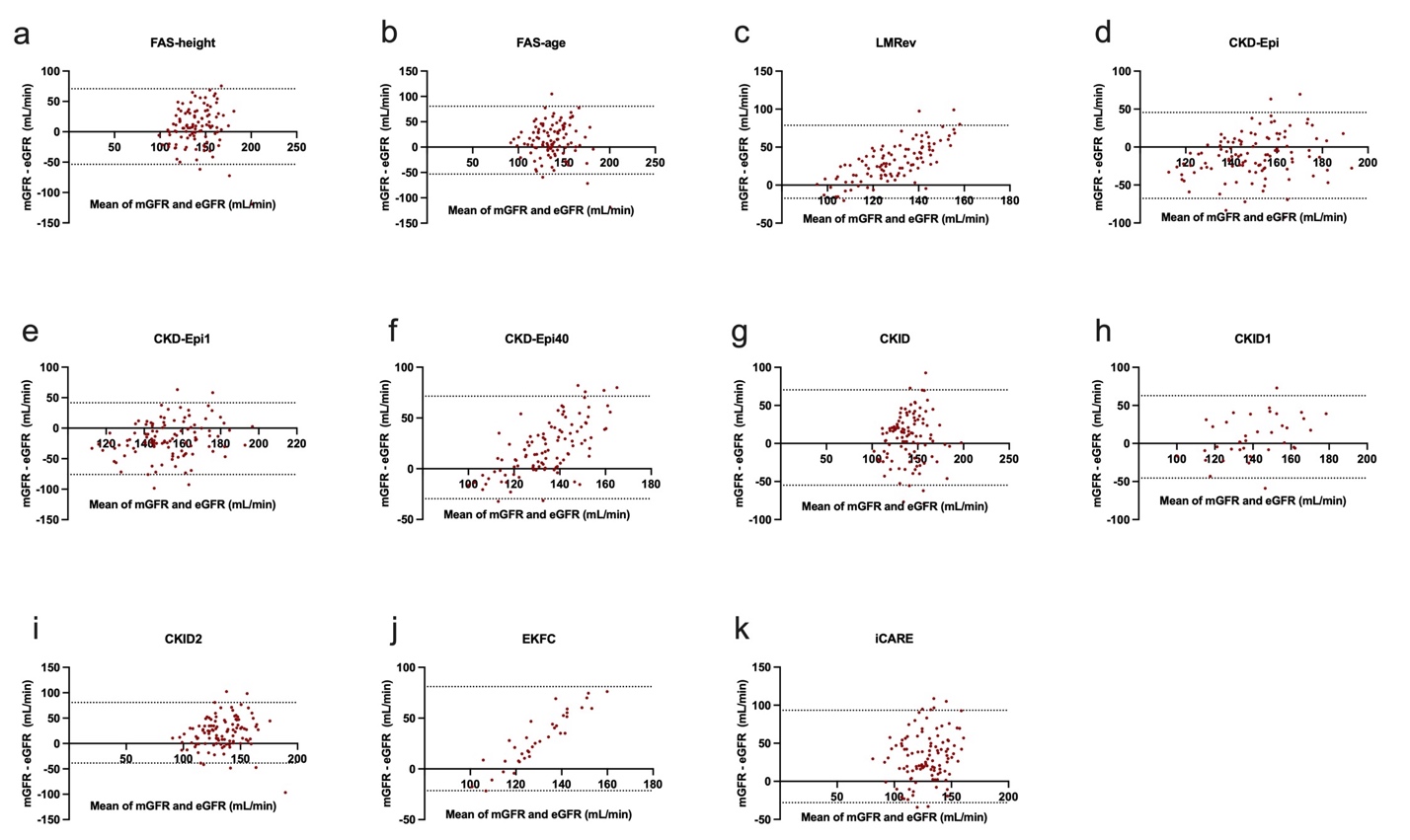


**Supplementary Figure S4.** Bland Altman plots for the <11 years subgroup

CDK-EPI: Chronic Kidney Disease Epidemiology; CKiD: Chronic Disease in Children; EKFC: European Kidney Function Consortium; FAS: Full-age spectrum; iCARE: Improving Renal Complications in Adolescents with Type 2 Diabetes through Research; LM-Rev: Lund-Malmo-revised; mGFR: measured glomerular filtration rate; eGFR: estimated glomerular filtration rat


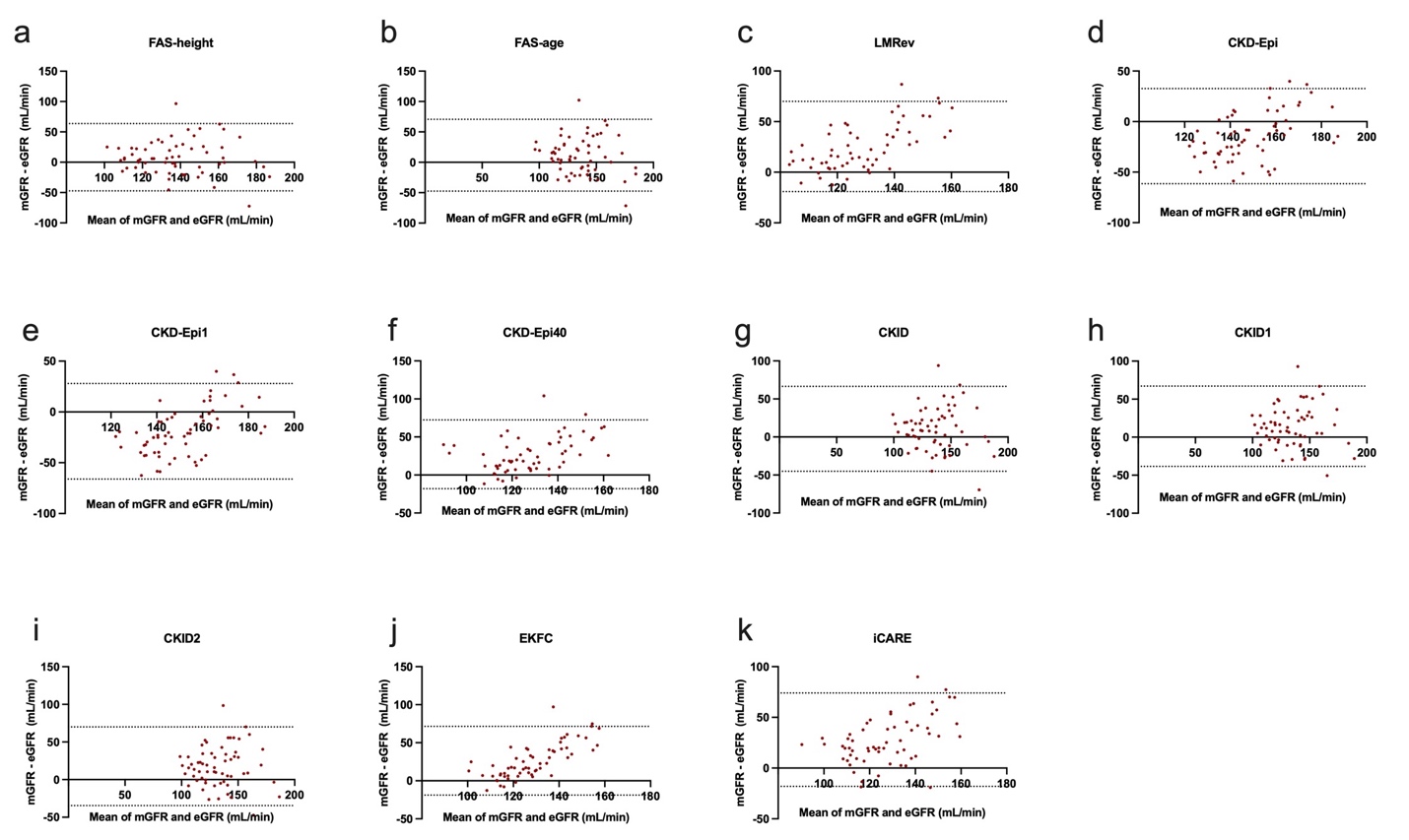
 **Supplementary Figure S5.** Bland Altman plots for the 11-15.9 years subgroup

CDK-EPI: Chronic Kidney Disease Epidemiology; CKiD: Chronic Disease in Children; EKFC: European Kidney Function Consortium; FAS: Full-age spectrum; iCARE: Improving Renal Complications in Adolescents with Type 2 Diabetes through Research; LM-Rev: Lund-Malmo-revised; mGFR: measured glomerular filtration rate; eGFR: estimated glomerular filtration rate


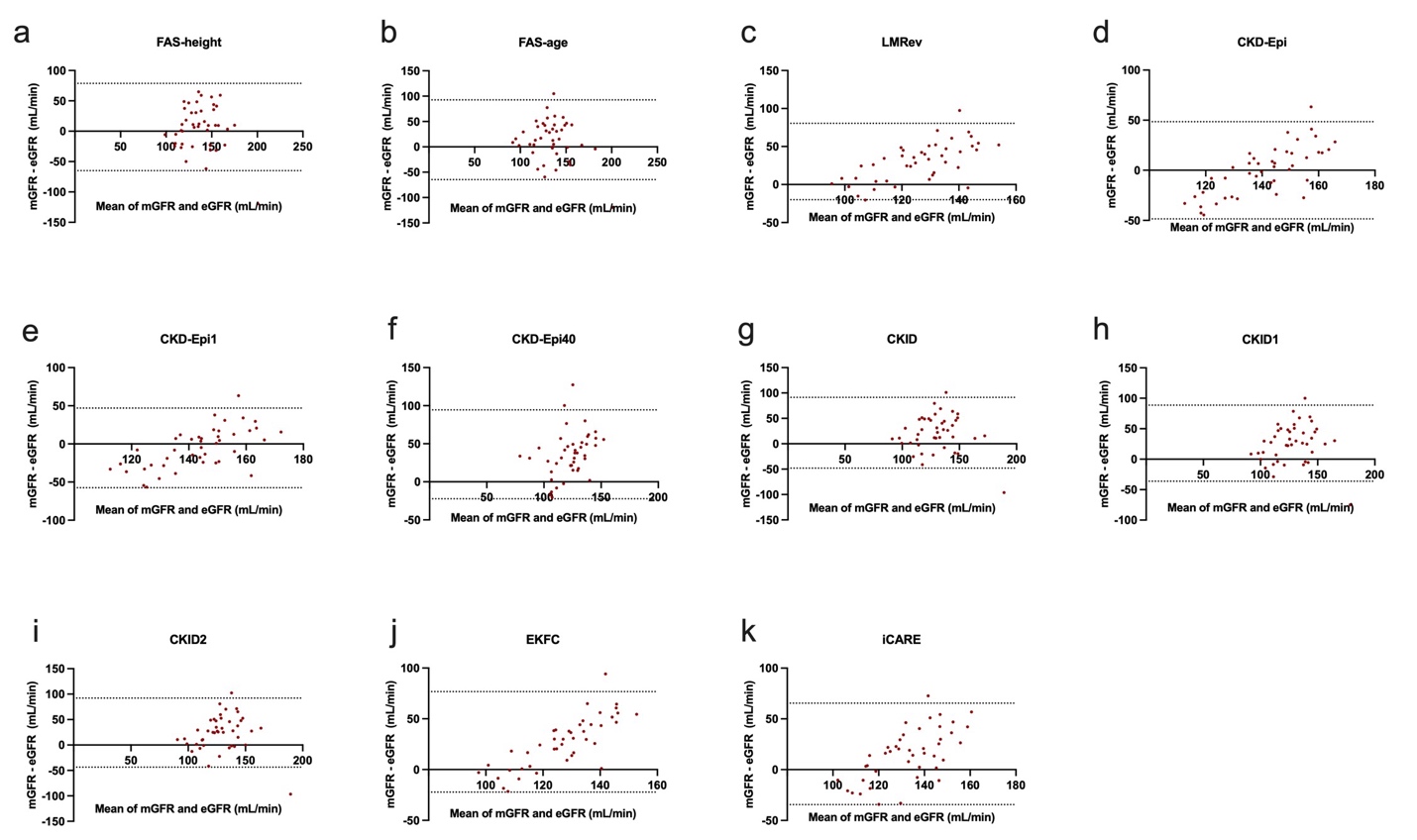


**Supplementary Figure S6.** Bland Altman plots for the 16-18. 5 years subgroup

CDK-EPI: Chronic Kidney Disease Epidemiology; CKiD: Chronic Disease in Children; EKFC: European Kidney Function Consortium; FAS: Full-age spectrum; iCARE: Improving Renal Complications in Adolescents with Type 2 Diabetes through Research; LM-Rev: Lund-Malmo-revised; mGFR: measured glomerular filtration rate; eGFR: estimated glomerular filtration rate
