# Supplementary material for: Which glomerular filtration rate estimation equations should be used in youth with type 1 diabetes?
Source: Pediatr Nephrol. 2025 Nov 15;41(3):801–8. doi: 10.1007/s00467-025-07057-w (PMC12852128; doi:10.1007/s00467-025-07057-w)
Supplement: Supplementary file 3 — (DOCX 30.6 KB) [file 467_2025_7057_MOESM3_ESM.docx]

**Supplementary Table S1. Equations for estimating of GFR evaluated in this study**

| Estimation method | Equation |
| --- | --- |
| FAS-height (Full Age Spectrum- Height) Equation [15] | eGFR = 107.3/creatinine/Q Q = 3.94–13.4 × height + 17.6 × height2−9.84 × L3+ 2.04 × height4 |
| FAS-Age (Full Age Spectrum – Age) Equation [16] | eGFR = 107.3/creatinine/Q Q = 0.0270 × age + 0.2329 |
| LM-Rev (revised Lund-Malmö) Equation  [17] | GFR = exp[X − 0.0158 × age + 0.438 × ln(age)] with X = 2.50 + 0.0121 × (150 − crea), for females and crea < 150 μmol/L X = 2.50 − 0.926 × ln(crea/150), for females and crea ≥ 150 μmol/L X = 2.56 + 0.00968 × (180 − crea), for males and crea < 180 μmol/L X = 2.56–0.926 × ln(crea/180), for males and crea ≥ 180 μmol/L |
| EKFC (European Kidney Function Consortium) Equation [18] | 107.3 × (SCr/Q)−0.322 [if SCr/Q <1]  107.3 × (SCr/Q)−1.132 [if SCr/Q ≥ 1] Males:  ln(Q) = 3.200 + 0.259 × *Age* − 0.543 × ln(*Age*) − 0.00763 × *Age*^2^ + 0.0000790 × *Age*^3^  Females:  ln(Q) = 3.080 + 0.177 × *Age* − 0.223 × ln(*Age*) − 0.00596 × *Age*^2^ + 0.0000686 × *Age*^3^ |
| CKD-EPI 2009 (Chronic Kidney Disease Epidemiology Collaboration, 2009) Equation [19] | eGFR = 141 x min {sCr/k,1}^α^ x max {sCr/k,1}^-1.209^ x 0.993^age^ x 1.108 [if female] x 1.159 [if black]  female: k = 61.8, α = -0.329  male: k = 79.6, α = -0.411 |
| CKD-EPI 2021(Revised CKD-EPI 2009 without race adjustment) Equation  [20] | Female: =141*MIN(sCr/61.8, 1)^-0.329 * MAX(sCr/61.8, 1)^-1.209 * 0.993^Age *1.108  Male: =141*MIN(sCr/79.6, 1)^-0.411 * MAX(sCr/79.6, 1)^-1.209 * 0.993^Age |
| CKD-EPI40 (Revised CKD-EPI 2021 with Age-Adjusted Creatinine) Equation  [10] | eGFR = 141 x min {sĈr/k,1}^α^ x max {sĈr/k,1}^-1.209^ x 0.993^40^ x 1.018 [if female] x 1.159 [if black]  male:  ln(sĈr) = ln(Cr) + 0.259 x (40- age) – 0.543 x ln(40/age) – 0.00763 x (40^2^ – age^2^) + 0.0000790 x (40^3^ – age^3^)  k = 79.3, α = -0.411  female:  ln(sĈr) = ln(Cr) + 0.177 x (40- age) – 0.223 x ln(40/age) – 0.00596 x (40^2^ – age^2^) + 0.0000686 x (40^3^ – age^3^)  k = 61.9, α = -0.329 |
| CKiD (Chronic Kidney Disease in Children) Equation [9] | eGFR = k x height/sCr  k = 41.3 |
| CKiD Under 25 (Sex-Dependent) Equation  [21] | eGFR = k x height/sCr  male: k = 41.8  female: k = 37.6 |
| CKiD Under 25 (Sex-and-Age-Dependent) Equation [21] | eGFR = k x height/sCr  males:  age 12 – 17 years: k = 39 x 1.045^(age – 12)^  age 18 – 25 years: k = 50.8  female:  age 12 – 17 years: k = 36.1 x 1.023^(age – 12)^  age 18 – 25 years: k – 41.4 |
| iCARE (Improving Renal Complications in Adolescents with Type 2 Diabetes through Research) Equation  [22] | Female: 50.7 x (BSA) ^0.186^ x (height/sCr)^0.405^ x 0.8994  Male: 50.7 x (BSA) ^0.186^ x (height/sCr)^0.405^ |
